# Publisher Correction: CryoEM structure of the type IVa pilus secretin required for natural competence in *Vibrio cholera*

**DOI:** 10.1038/s41467-020-19389-2

**Published:** 2020-10-27

**Authors:** Sara J. Weaver, Davi R. Ortega, Matthew H. Sazinsky, Triana N. Dalia, Ankur B. Dalia, Grant J. Jensen

**Affiliations:** 1 Division of Chemistry and Chemical Engineering, California Institute of Technology, 1200 E. California Blvd, Pasadena, CA 91125 USA; 2 Division of Biology and Biological Engineering and Howard Hughes Medical Institute, California Institute of Technology, 1200 E. California Blvd, Pasadena, CA 91125 USA; 3 Department of Chemistry, Pomona College, 333N. College Way, Claremont, CA 91711 USA; 4 Department of Biology, Indiana University, 107S. Indiana Avenue, Bloomington, IN 47405 USA; 5 Present Address: Howard Hughes Medical Institute, David Geffen School of Medicine, Departments of Biological Chemistry and Physiology, University of California Los Angeles, 615 Charles E Young Drive South, Los Angeles, CA 90095 USA

**Keywords:** Bacterial structural biology, Cryoelectron microscopy

Correction to: *Nature Communications* 10.1038/s41467-020-18866-y, published online 8 October 2020.

The original version of this Article contained an error in the author affiliations.

The affiliation of Grant Jensen with Division of Biology and Biological Engineering and Howard Hughes Medical Institute, California Institute of Technology, 1200 E. California Blvd, Pasadena, CA 91125, USA was inadvertently omitted. Grant Jensen was incorrectly associated with Howard Hughes Medical Institute, David Geffen School of Medicine, Departments of Biological Chemistry and Physiology, University of California Los Angeles, 615 Charles E Young Drive South, Los Angeles, CA 90095, USA.

This has now been corrected in both the PDF and HTML versions of the Article.

