# Supplementary material for: CryoEM structure of the type IVa pilus secretin required for natural competence in Vibrio cholerae
Source: Nat Commun. 2020 Oct 8;11:5080. doi: 10.1038/s41467-020-18866-y (PMC7545093; doi:10.1038/s41467-020-18866-y)
Supplement: Supplementary file 1 — Supplementary Information [file 41467_2020_18866_MOESM1_ESM.pdf]

Supplementary information

CryoEM Structure of the Type IVa Pilus Secretin Required for Natural Competence in *Vibrio cholerae*

Sara Weaver et al.

Sara J. Weaver[1,5] , Davi R. Ortega[2], Matthew H. Sazinsky[3], Triana N. Dalia[4], Ankur B. Dalia[4], Grant J. Jensen[2,6]

[1]Division of Chemistry and Chemical Engineering, California Institute of Technology, 1200 E. California Blvd, Pasadena, CA 91125, USA

[2]Division of Biology and Biological Engineering and Howard Hughes Medical Institute, California Institute of Technology, 1200 E. California Blvd, Pasadena, CA 91125

[3]Department of Chemistry, Pomona College, 333 N. College Way, Claremont, CA 91711, USA

[4]Department of Biology, Indiana University, 107 S. Indiana Avenue, Bloomington, IN 47405, USA

[5]Present address Howard Hughes Medical Institute, David Geffen School of Medicine, Biological Chemistry, University of California, 615 Charles E Young Drive South, Los Angeles CA 90095, USA,

[6]Corresponding author email jensen@caltech.edu

Supplemental Figure 1

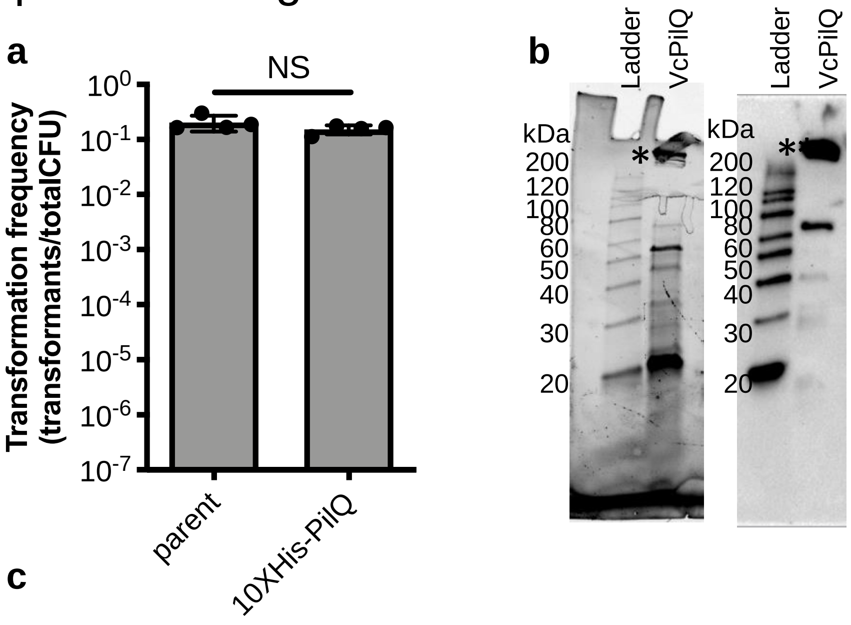

c

```
001   MKNGLKTYVAQTWLTLWVGLALCASSMVFSAESATANQLENIDFRVNKEK
051   AAVLIVELASPSAVVDVQKVQEGLSIELLKTDVADDKLYLLDVKDFSTPV
101   ESVEVFRKEPSTQLVVTVDGEFQHDYTLKGKYLEVVISKLKADEKPKPKS
151   VLEKEGKLISINFQDIPVRNVLQLIADYNGFNLVVSDSVVGNLTLRLDGV
201   PWQQVLDIILQVKGLDKRVDGNVILIAPKEELDLREKQALEKARLAEELG
251   DLKSEIIKINFAKASDIAAMIGGEGNVNMLSERGSISIDERTNSLLIREL
301   PDNIAVIREIIESLDIPVKQVQIEARIVTVKEGNLEELGVRWGVMSTNGS
351   HSVGGSIESNLWQKGLLADDEFPVDEFLNVNLASTSANASSIAFQVAKLG
401   SGTLLDLELSALQNESKAEIISSPRLITTNKQPAYIEQGTEIPYLESSSS
451   GASTVAFKKAVLSLKVTPQITPDNRLVLDLSVTQDRRGETVKTGTGEAVS
501   IDTQRIGTQVLVNNGETVVLGGIFQHSINNSVDKVPLLGDLPVLGALFRR
551   TYEQMGKSELLIFVTPKVVIQ
```

Bold residues (as printed): 051–100 all; 101 **ESVEVFRKEPSTQLVVTVDGEFQHDYTLK**GK**YLEVVISKLK**ADEKPKPK**S**; 151 **VLEKEGKLISINFQDIPVR**…**LDGV**; 201 **PWQQVLDIILQVKGLDKRVDGNVILIAPKEELDLREK**QALEK**ARLAEELG**; 251–300 all; 301 **PDNIAVIREIIESLDIPVKQVQIEARIVTVKEGNLEELGVR**WGVMSTNGS; 351 HSVGGSIESNLWQK**GLLADDEFPVDEFLNVNLASTSANASSIAFQVAKLG**; 401 **SGTLLDLELSALQNESKAEIISSPR**…; 451 GASTVAFK**KAVLSLKVTPQITPDNRLVLDLSVTQDRRGETVKTGTGEAVS**; 501 **IDTQR**…; 551 **TYEQMGKSELLIFVTPK**VVIQ

Supplemental Figure 1: Expression and purification of VcPilQ from *V. cholerae* cells

(a) The functionality of N-terminally deca-histidine tagged native *Vibrio cholerae* PilQ (VcPilQ, strain TND1751) was assessed via natural transformation assays. The Y axis displays Transformation frequency in units of transformants per total colony forming units (CFUs). These data indicate that the tagged variant supports equal rates of transformation compared to an isogenic parent strain, thus indicating that it is fully functional. Data are from four independent biological replicates and shown as the mean ± standard deviation. Statistical comparisons were made by unpaired Student's t-test. NS = not significant. The raw data are available in Source Data.

(b) Representative gel electrophoresis analysis of VcPilQ in amphipols by stain-free exposure (left) and western blotting against the histidine tag with anti-His antibody (right). The multimer band is indicated with an asterisk. The ladder bands are labeled in units of kiloDalton (kDa). The raw data are available in Source Data.

(c) The VcPilQ amino acid sequence is shown with the coverage achieved by mass spectrometry is represented by bolded residues in the sequence. The lefthand column shows the sequence index of the first residue of each line. Approximately 65% of the sequence was represented in the peptides analyzed by mass spectrometry. The full mass spectrometry results are available in Source Data.

Supplemental Figure 2

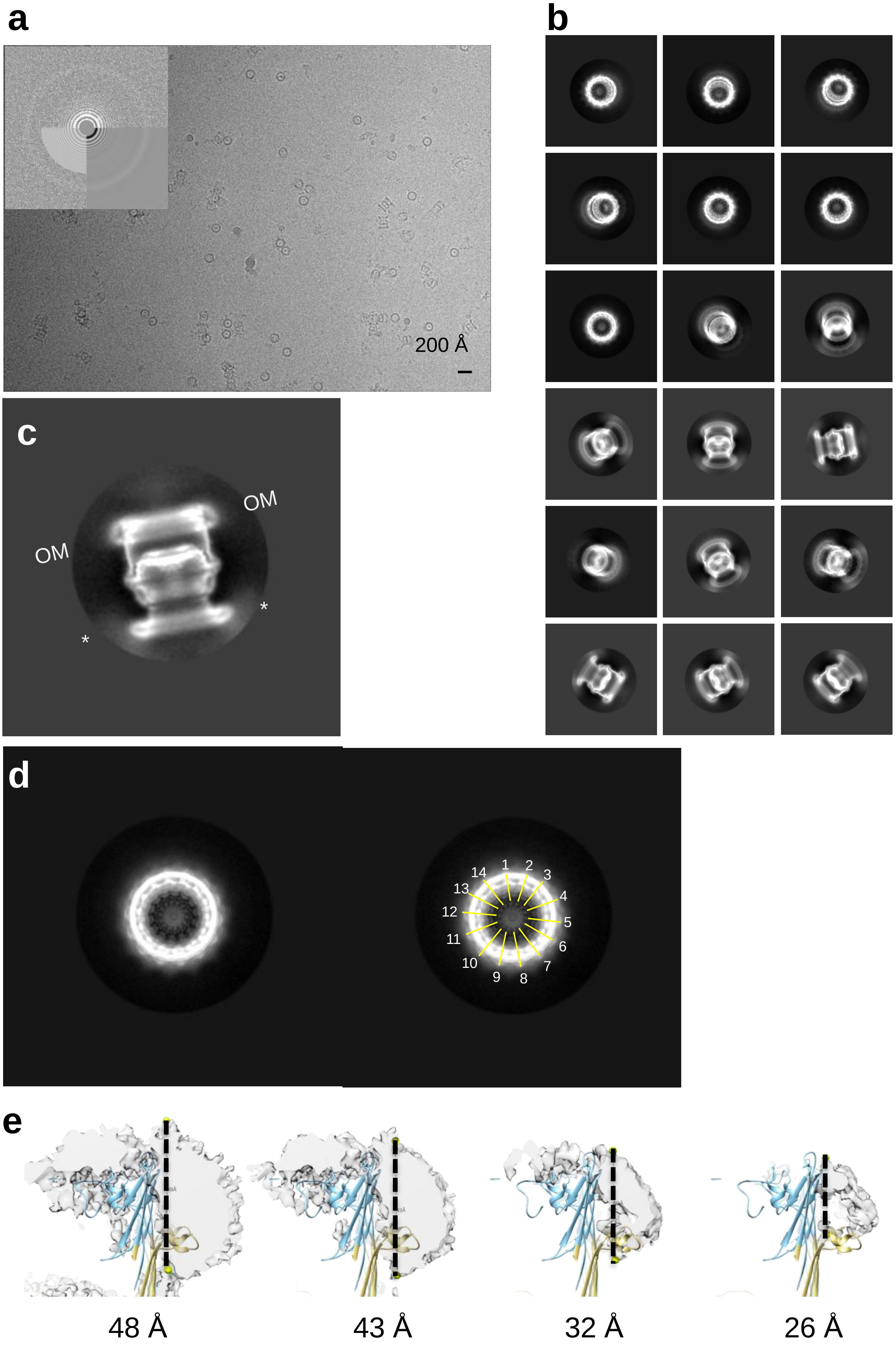

Supplemental Figure 2: VcPilQ CryoEM data

(a) Representative micrograph with a 10 Å low pass filter applied. A representative Fourier transform and contrast transfer function (CTF) fit of the micrograph is shown in the upper left corner. Scale bar, 200 Ångstrom.

(b) Representative 2D classes calculated in Relion. The 2D class box is 441.6 Å wide.

(c) A 2D class of *Vibrio cholerae* PilQ (VcPilQ) is shown with the outer membrane (OM) labeled. Asterisks indicate fuzzy density which we speculate could represent the AMIN domain.

(d) A top view 2D class (left) demonstrates that VcPilQ has C14 symmetry. On the right, the class average is shown with yellow lines point to inner spokes in the PilQ gate. Each monomer is numbered in white.

(e) VcPilQ micelle density with putative outer membrane diameter labeled in Å. The non-protein cryoEM density of VcPilQ is shown in grey at four different Chimera thresholds (left to right). The atomic model of VcPilQ is shown in blue and yellow according to the color scheme of Figure 1. In each image, yellow spheres mark the approximate top and bottom of the micelle density. The distance between the yellow spheres (dashed line) is shown below each image in Ångstroms.

Supplemental Figure 3

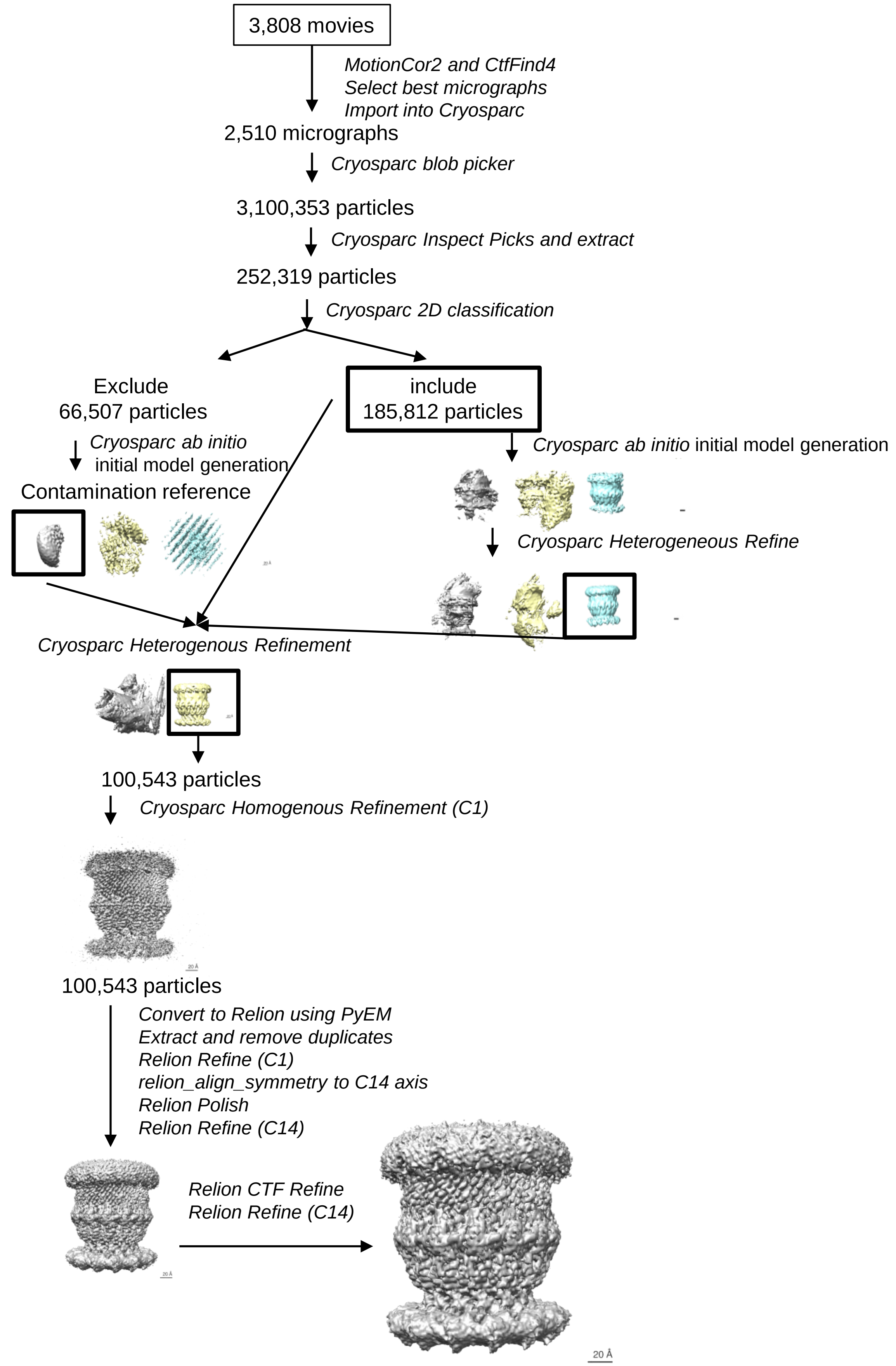

Supplemental Figure 3: CryoEM data processing flow chart

The major steps in data processing are summarized in a flow chart. Full processing details are available in Methods. The text in italics describes actions taken between each step. Images represent the 3D reconstructions generated as output for different programs. Within a step, the colors delineate different outputs. If an image is boxed, it means it was used as input for the next step.

Supplemental Figure 4

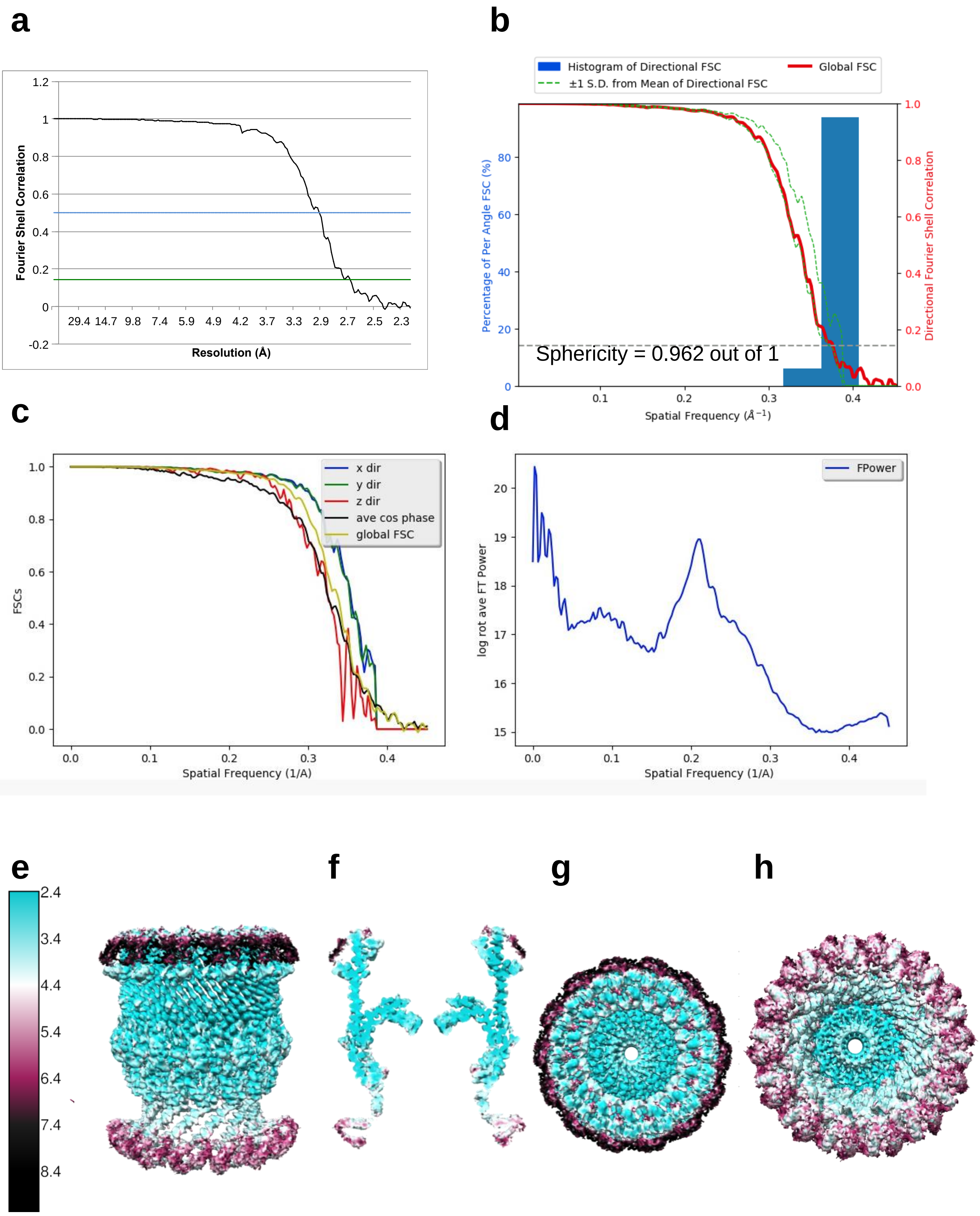

Supplemental Figure 4: Fourier Shell Correlation, 3DFSC, and local resolution of VcPilQ

(a) The Fourier Shell Correlation (FSC) of the *Vibrio cholerae* PilQ (VcPilQ) cryoEM structure was calculated in Relion_postprocess. The 0.143 threshold (green line) suggests 2.7 Å resolution while the 0.5 threshold (blue line) suggests 3.0 Å resolution.

(b) to (d) The Salk Institute Three Dimensional Fourier Shell Correlation (3DFSC) server was used to examine the resolution in the X, Y and Z dimensions in the VcPilQ structure[1]. The sphericity is 0.962 out of 1. In (b), the X-axis is spatial frequency (1/Å) and the histogram of the directional FSC is plotted in blue as percentage of per angle FSC, while the Global FSC is plotted against a Y axis with units of Directional FSC. The plus or minus one standard deviation of the mean of the directional FSC is plotted with a dashed green line. In (c), the FSC of the x direction (blue), the y direction (green), the z direction (red), the average cosine phase (black) and the global FSC (gold) are plotted against spatial frequency (1/Å). In (d), the Fourier Power (FPower) is plotted in blue against spatial frequency (1/Å) in units of log of the rotational average Fourier Transform Power.

(e-h) ResMap was used to calculate the local resolution per voxel of the density map. Resolution is visualized with a color gradient ranging from 2.4 Å (cyan) to 7.4 Å (maroon) to 8.4 Å (black). VcPilQ is shown from the side (e), as a central slice (f), from the top (extracellular side, g) and from the bottom (periplasmic side, h).

Supplemental Figure 5

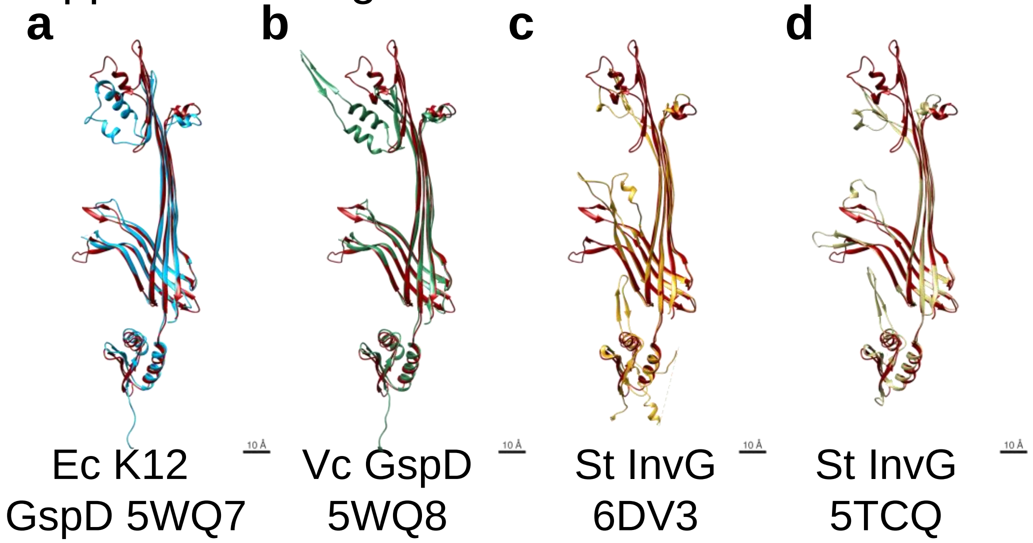

e

VcPilQ Compared to	RMSD (Å)
E. coli K12 GspD (5WQ7)	1.2
V. cholerae GspD (5WQ8)	1.2
S. typhimurium InvG (6DV3)	0.97
S. typhimurium InvG (5TCQ)	1.1

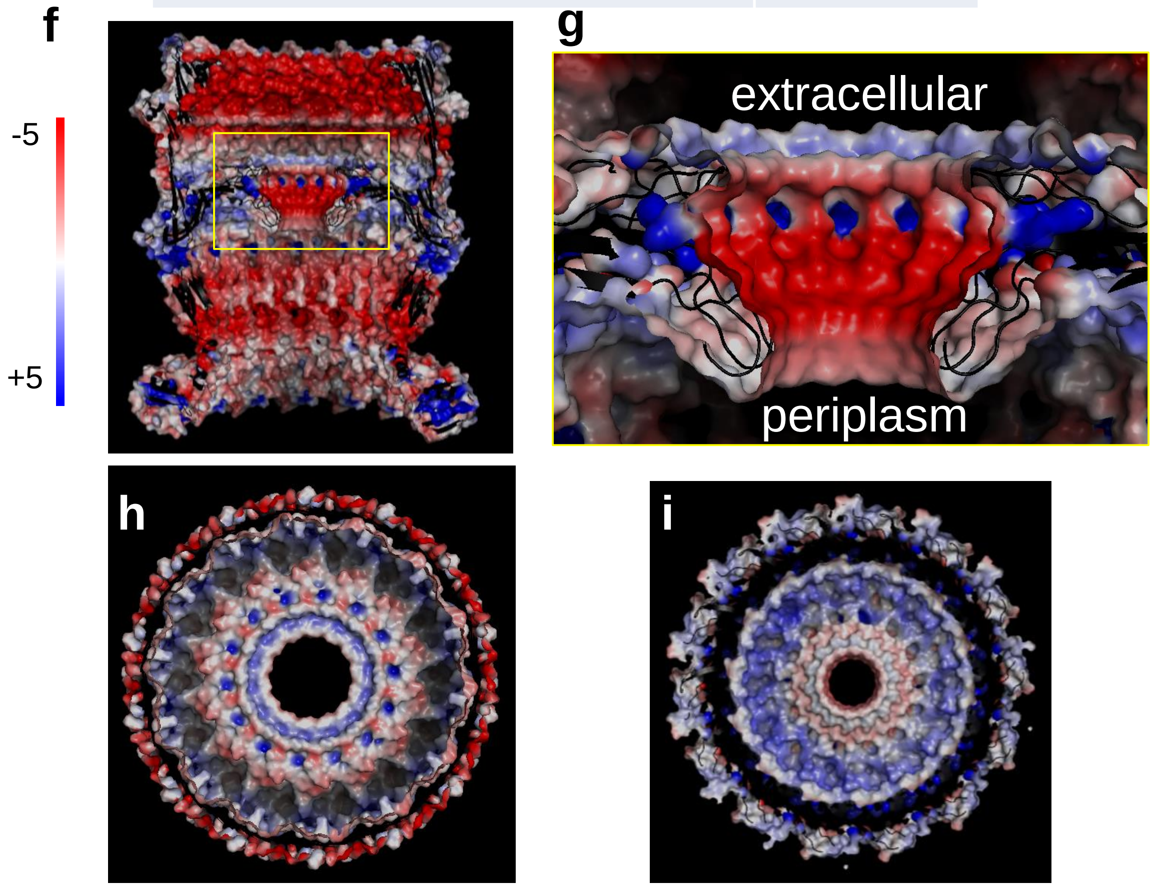

Supplemental Figure 5: RMSD of VcPilQ versus published secretin structures.

The monomer structure of *Vibrio cholerae* PilQ (VcPilQ) (dark red) was aligned to the *E. coli* K12 GspD (EcGspD, PDB 5WQ7) (a), the *V. cholerae* GspD (VcGspD, PDB 5WQ8) (b), and the *S. typhimurium* InvG (StInvG, PDB 6DV3 and 5TCQ) (c-d)[2–4]. Only the N3 and Secretin domains were compared. Root-mean-square deviation (RMSD) was calculated with MatchMaker in Chimera. The RMSD results are summarized in a Table (e).

(f-i) In Pymol, the Adaptive Poisson-Boltzmann Solver was used to calculate the electrostatic potential calculation of VcPilQ[5]. The scale varies from -5 (red) to +5 (blue) in units of K_bT/e_c. In (f) the inner cavity of VcPilQ is shown. The yellow box in (f) is blown up in (g). End-on views of VcPilQ from the extracellular side (h) and the periplasmic side (i) are shown.

Supplemental Figure 6

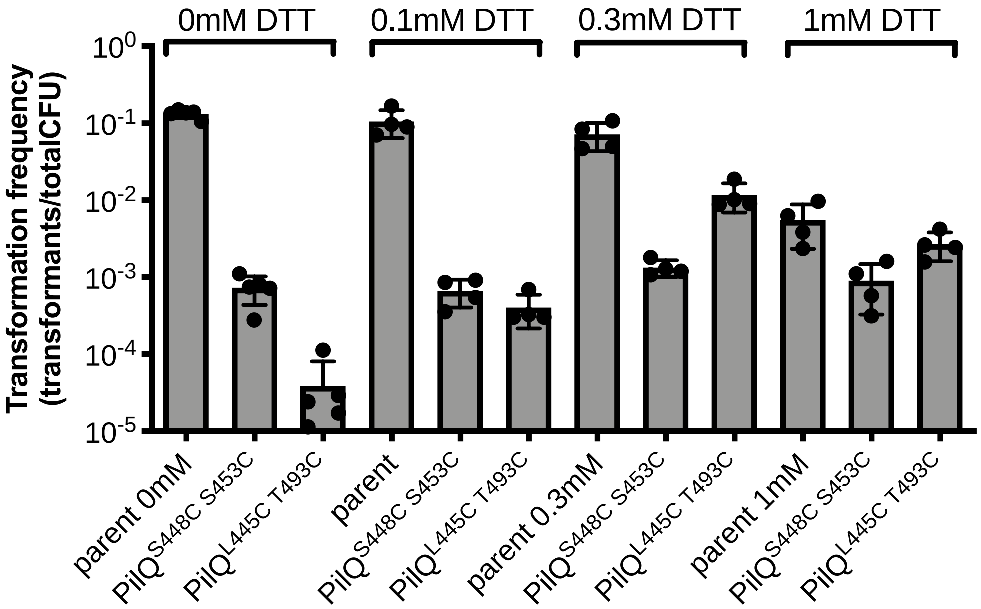

Supplemental Figure 6: Raw transformation frequency data for cysteine pair mutants.

The Transformation assays in Figure 3D were performed with the indicated strains (parent=TND2140, $PilQ^{S448C\ S453C}$=TND2169, $PilQ^{L445C\ T493C}$=TND2170) in the presence of 0 to 1 mM dithiothreitol (DTT) as indicated above the bars. Data are from at least 4 independent biological replicates and shown as the mean ± standard deviation. The Y axis displays Transformation frequency in units of transformants per total colony forming units (CFUs). Raw data are available in the Source Data.

Supplemental Figure 7

a

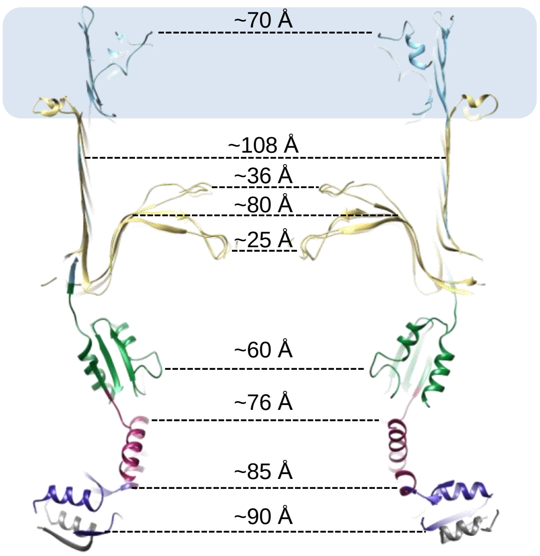

b

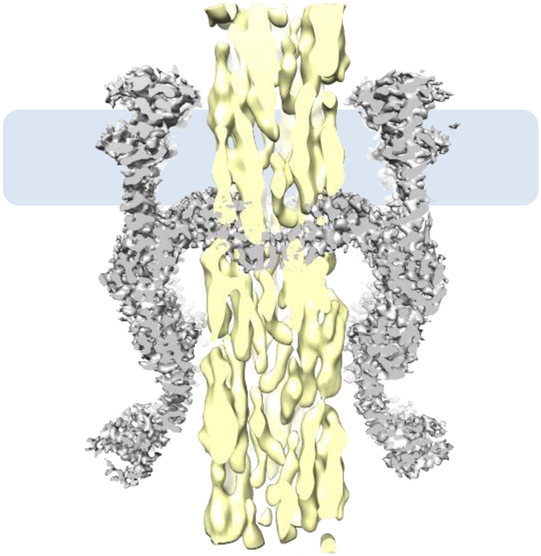

Supplemental Figure 7: VcPilQ inner channel distances and comparison of VcPilQ with the *E. coli* Type IVa pilus

(A) A central slice of the atomic model of *Vibrio cholerae* PilQ (VcPilQ) colored as in Figure 1. The inner cavity distances are depicted with dashed lines and labeled in ångstroms. The outer membrane is depicted as a blue rectangle.

(B) A central slice of the cryoEM density of VcPilQ (grey) is shown with the *E. coli* Type IVa pilus structure (EMD-0070, yellow) fit in[6]. The outer membrane is depicted as a blue rectangle.

Supplementary Table 1: Strain List

Strain name in article	Genotype and antibiotic resistance(s)	Description	Experiment	Parent strain	Source
El Tor strain E7946	E7946 Sm^R		Source	n/a	Ref. 7
TND1751	10xHis-PilQ, ΔVC1807::SpecR, lacZ::lacIq, comEA-mCherry, ΔluxO, Ptac-tfoX, ΔTCP::ZeoR, ΔMSHA::CarbR, ΔCTX::KanR	His-tagged PilQ at native locus	Protein purification and natural transformation assay (Figure 1 and Supplemental Figure 1)	El Tor strain E7946	This paper
TND2140	ΔlacZ::Pbad-10XHis-PilQ CmR, ΔpilQ::TetR, ΔCTX::KanR, ΔMSHA::CarbR, ΔluxO, ΔTCP::ZeoR, comEA-mCherry, Ptac-tfoX	His-tagged PilQ expressed at ectopic site	Natural transformation assay (Figure 3d and Supplemental Figure 6)	El Tor strain E7946	This paper
TND2169	ΔlacZ::Pbad-10XHis-PilQ S448C S453C CmR, ΔpilQ::TetR, ΔCTX::KanR, ΔMSHA::CarbR, ΔluxO, ΔTCP::ZeoR, comEA-mCherry, Ptac-tfoX	His-tagged PilQ (S448C S453C) expressed at ectopic site	Natural transformation assay (Figure 3d and Supplemental Figure 6)	TND2140	This paper
TND2170	ΔlacZ::Pbad-10XHis-PilQ L445C T493C CmR, ΔpilQ::TetR, ΔCTX::KanR, ΔMSHA::CarbR, ΔluxO, ΔTCP::ZeoR, comEA-mCherry, Ptac-tfoX	His-tagged PilQ (L445C T493C) expressed at ectopic site	Natural transformation assay (Figure 3d and Supplemental Figure 6)	TND2140	This paper
TND2244	ΔlacZ::Pbad-10XHis-PilQ CmR, ΔpilT::TmR, ΔpilQ::TetR, ΔCTX::KanR, ΔMSHA::CarbR, ΔluxO, ΔTCP::ZeoR, pilA S67C, comEA-mCherry, Ptac-tfoX	Hyperpiliated strain (ΔpilT) for labeling pili (pilA S67C) with His-tagged PilQ expressed at ectopic site	Surface piliation assay (Figure 3e-g)	Ellison *et al*. 2018	This paper
TND2242	ΔlacZ::Pbad-10XHis-PilQ S448C S453C CmR, ΔpilT::TmR, ΔpilQ::TetR, ΔCTX::KanR, ΔMSHA::CarbR, ΔluxO, ΔTCP::ZeoR, pilA S67C, comEA-mCherry, Ptac-tfoX	Hyperpiliated strain (ΔpilT) for labeling pili (pilA S67C) with His-tagged PilQ (S448C S453C) expressed at ectopic site	Surface piliation assay (Figure 3e-g)	TND2244	This paper
TND2243	ΔlacZ::Pbad-10XHis-PilQ L445C T493C CmR, ΔpilT::TmR, ΔpilQ::TetR, ΔCTX::KanR, ΔMSHA::CarbR, ΔluxO, ΔTCP::ZeoR, pilA S67C, comEA-mCherry, Ptac-tfoX	Hyperpiliated strain (ΔpilT) for labeling pili (pilA S67C) with His-tagged PilQ (L445C T493C) expressed at ectopic site	Surface piliation assay (Figure 3e-g)	TND2244	This paper

Supplementary Table 2: Primers

Primer Name	Sequence (5’ → 3’)	Description
DOG0436	AAGCGTTCTGGTTCAATCACC	ΔMSHA F1 (UP arm)
DOG0434	gtcgacggatccccggaatCATTCTCTACCACTGCTATTTGGTTC	ΔMSHA R1 (UP arm)
BBC2129	gaagcagctccagcctacaTAATGATTAAGCACTCAATGGATCCAG	ΔMSHA F2 (DOWN arm)
BBC2130	TTTTGCATCAGCAAAATCACGC	ΔMSHA R2 (DOWN arm)
BBC2123	TGATGGTAACTACTATAGGGTCG	ΔTCP F1 (UP arm)
BBC2124	gtcgacggatccccggaatTAAATTAGGCTAGTGCCAGTCAG	ΔTCP R1 (UP arm)
BBC2125	gaagcagctccagcctacaACAGGAGTTGCAGAAAAATAATGG	ΔTCP F2 (DOWN arm)
BBC2126	ACTAAGATAATTGCTTCACGCATG	ΔTCP R2 (DOWN arm)
BBC1443	GATTCAGACGGCAACTATCG	ΔCTX F1 (UP arm)
BBC1444	gtcgacggatccccggaatGCGATTACACCATCAATCC	ΔCTX R1 (UP arm)
BBC1445	gaagcagctccagcctacaGCACTAGGAACATTTTGTCTC	ΔCTX F2 (DOWN arm)
BBC1447	CTGAAATGTGCTGTGTAAGC	ΔCTX R2 (DOWN arm)
BBC374	TGGCAAAAAGCGAGAGAAGAAG	Δ*luxO* F1 (UP arm)
BBC375	gtcgacggatccccggaatCATGAGGACATATTTTGTTTTCTGC	Δ*luxO* R1 (UP arm)
BBC376	gaagcagctccagcctacaTAAGCGATGAGAGAATGGATCAAC	Δ*luxO* F2 (DOWN arm)
BBC377	TCACACCCGAATTTCCATCATGC	Δ*luxO* R2 (DOWN arm)
BBC1876	CGTTGTACCATGCATCTTTTGAG	ΔpilQ F1 (UP arm)
BBC1877	gtcgacggatccccggaatCATGAGGCTAAGCTCCAGTAAAG	ΔpilQ R1 (UP arm)
BBC1878	gaagcagctccagcctacaTAACTTAGCGTGTGCGTAAC	ΔpilQ F2 (DOWN arm)
BBC1879	GTACCAACATCCACAATTCGG	ΔpilQ R2 (DOWN arm)
DOG0400	ACTTCTGGCTGAAGGTCAATTTTC	Δ*pilT* F1 (UP arm)
DOG0401	gtcgacggatccccggaatCATTTAAATTCCTTAATAAAGTCTGGC	Δ*pilT* R1 (UP arm)
DOG0402	gaagcagctccagcctacaTAGGTAGGTAAAGACAGATGGAG	Δ*pilT* F2 (DOWN arm)
DOG0403	TCACGTGTTCGGCCAAAATC	Δ*pilT* R2 (DOWN arm)
DOG0582	ACATCTGGCAGATCTGATTTCAC	10XHis PilQ F1 (UP arm)
BBC2888	tccaccacttccacctgcGGCGGTAGCGGATTCAGCAC	10XHis PilQ R1 (UP arm)
BBC2892	gcaggtggaagtggtggaCATCATCACCATCATCATCATCACCATCACgcaggtggagcaggtgga	10XHis MIDDLE F

**Primer Name**	**Sequence (5' → 3')**	**Description**
BBC2893	tccacctgctccacctgcGTGATGGTGATGATGATGATGGTGATGATGtccaccacttccacctgc	10XHis MIDDLE R
BBC2889	gcaggtggagcaggtggaAACCAGTTGGAAAACATCGAC	10XHis PilQ F2 (DOWN arm)
BBC1907	TTCGTCCAATGCGTACAAGG	10XHis PilQ R2 (DOWN arm)
BBC1966	caatttcacacaggatcccgggAGGAGGTATAACGATGAAAAATGGATTGAAAACTTATG	Amplify PilQ for Pbad construct F
BBC2027	tgtaggctggagctgcttcTTATTGAATGACCACTTTCGGC	Amplify PilQ for Pbad construct R
BBC3128	CGCGACCGTgcAGGCACCACTGGAgcACGATTCCAGATAAGGG	PilQ S448C S453C R1
BBC3129	CCCTTATCTGGAATCGTgcTCCAGTGGTGCCTgcACGGTCGCG	PilQ S448C S453C F2
BBC3131	CTGGAAGACGATTCacaATAAGGGATCTCAG	PilQ L445C T493C R1
BBC3132	CTGAGATCCCTTATtgtGAATCGTCTTCCAG	PilQ L445C T493C F2

*Lower case demarcates overlap sequences, or the nucleotides mutated for site directed mutants.

Supplementary Data 1: Complete tree of Proteobacteria T4aP Secretins, domain architecture and gene neighborhood.

The complete tree of 197 representative sequences of Type IVa Pilus (T4aP) Secretins collected from Proteobacteria is shown (left) with the domain architecture (center) and the gene neighborhood (right). The tree is summarized in Figure 4a and a branch is featured in Figure 4b. The species name and locus number are provided for each sequence. Sequences boxed in dark green are representatives from the genera shown to perform natural transformation (*Haemophilus, Moraxella, Neisseria, Pseudomonas,* and *Vibrio*). For each sequence (represented as a thick grey line), the domain architecture was identified using the Comprehensive Domain Visualization Tool (CD-VIST) pipeline. Domains were identified using HMMER (a Hidden Markov Model package) (black outline), HHblits (HMM-HMM–based lightning-fast iterative sequence search) (red outline) or TMHMM (Transmembrane Hidden Markov Model) (grey shaded rectangle) for transmembrane regions. Domains are identified by their PFAM name. In the right column, GeneHood was used to depict the gene neighborhood of the Secretin sequence as a series of arrows, where the direction indicates orientation. A color-coded key for the genes discussed in the paper is shown at the bottom, from left to right, the key highlights the *VC2641* (light red), *VC2640* (light red-orange), *VC2638* (light orange), *VC2637* (light yellow), *VC2636* (light green), *mcrA* (red), *pilM* (orange), *pilN* (golden), *pilO* (golden), *pilP* (green), *pilQ* (teal), *aroKB* (aqua blue), *VC2627* (cerulean blue), *VC2626* (royal blue), *VC2625* (purple), *VC2624* (magenta), *VC2623* (pink-red), *VC2622* (seafoam green), *VC2621* (light teal), and *VC2620* (light blue).

Supplementary Data 2: Sequences and alignment of Proteobacteria T4aP Secretins. Instructions on how to parse the data are available at https://gitlab.com/jensenlab/seccomp.

Supplementary Data 3: GeneHood Analysis. A json.gz file containing the GeneHood analysis that can be visualized at https://next.genehood.io/.

References

1. Tan, Y. *et al.* Addressing preferred specimen orientation in single-particle cryo-EM through tilting. *Nature Methods* **14**, 793–796 (2017).
2. Worrall, L. *et al.* Near-atomic-resolution cryo-EM analysis of the Salmonella T3S injectisome basal body. *Nature* **540**, 597 (2016).
3. Yan, Z., Yin, M., Xu, D., Zhu, Y. & Li, X. Structural insights into the secretin translocation channel in the type II secretion system. *Nature Structural & Molecular Biology* **24**, 177–183 (2017).
4. Hu, J. *et al.* Cryo-EM analysis of the T3S injectisome reveals the structure of the needle and open secretin. *Nat Commun* **9**, 1–11 (2018).
5. Jurrus, E. *et al.* Improvements to the APBS biomolecular solvation software suite. *Protein Science* **27**, 112–128 (2018).
6. Bardiaux, B. *et al.* Structure and Assembly of the Enterohemorrhagic Escherichia coli Type 4 Pilus. *Structure* **27**, 1082-1093.e5 (2019).
7. Miller, V. L., DiRita, V. J. & Mekalanos, J. J. Identification of toxS, a regulatory gene whose product enhances toxR-mediated activation of the cholera toxin promoter. *J. Bacteriol.* **171**, 1288–1293 (1989).
