# Supplementary figures and images for: CryoEM structure of the type IVa pilus secretin required for natural competence in Vibrio cholerae
Source: Nat Commun. 2020 Oct 8;11:5080. doi: 10.1038/s41467-020-18866-y (PMC7545093; doi:10.1038/s41467-020-18866-y)

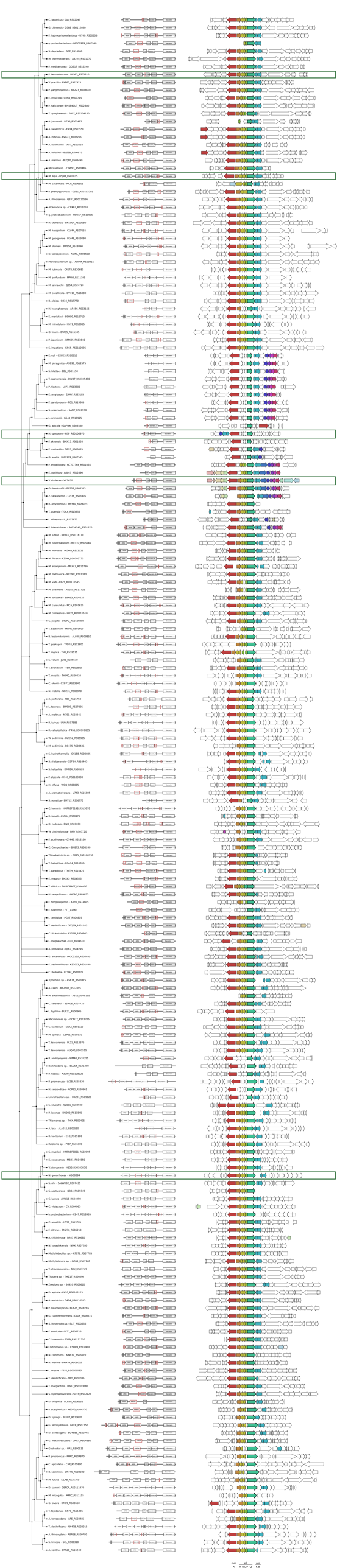

Supplement: Supplementary file 3 — Supplementary Data set 1 [file 41467_2020_18866_MOESM3_ESM.zip › Supplementary Data 1.pdf]
